# Phenotyping the Use of Cangrelor in Percutaneous Coronary Interventions

**DOI:** 10.3390/ph18030432

**Published:** 2025-03-19

**Authors:** Nikolaos Pyrpyris, Kyriakos Dimitriadis, Konstantinos G. Kyriakoulis, Stergios Soulaidopoulos, Panagiotis Tsioufis, Aggelos Papanikolaou, Nikolaos G. Baikoussis, Alexios Antonopoulos, Konstantinos Aznaouridis, Konstantinos Tsioufis

**Affiliations:** 1 First Department of Cardiology, School of Medicine, National and Kapodistrian University of Athens, Hippokration General Hospital, 115 27 Athens, Greece; npyrpyris@gmail.com (N.P.); konkyriakoulis@gmail.com (K.G.K.); soulaidopoulos@hotmail.com (S.S.); agepap25@otenet.gr (A.P.); antonopoulosal@yahoo.gr (A.A.); conazna@yahoo.com (K.A.); ktsioufis@gmail.com (K.T.); 2 Department of Cardiac Surgery, Hippokration General Hospital, 115 27 Athens, Greece; nikolaos.baikoussis@gmail.com

**Keywords:** cangrelor, antiplatelet, percutaneous coronary intervention, cardiogenic shock, microcirculation, coronary artery disease

## Abstract

The use of antiplatelet agents is essential in percutaneous coronary interventions, both periprocedurally and in the post-interventional period. Procedural antiplatelet therapy, aiming to limit ischemic complications, is mostly administered with oral agents, including aspirin and P2Y12 inhibitors. However, there are several limitations in the use of oral P2Y12 inhibitors, including their difficult administration in patients presenting with cardiogenic shock and their relatively slower onset of action, leaving a significant period of the procedure with a suboptimal antiplatelet effect. These pitfalls could be avoided with the use of cangrelor, the only available intravenous P2Y12 inhibitor, which has a rapid onset and offset antiplatelet effect, as well as a favorable pharmacological profile. The use of cangrelor has been increasing in recent years, with several studies aiming to determine what the optimal patient phenotype to receive such treatment ultimately is and how its use could be adjunctive to oral P2Y12 inhibitors. Therefore, the aim of this review is to provide an overview of the pharmacological profile of cangrelor and an update regarding the clinical evidence supporting its use, as well as to discuss the optimal patient phenotype, related clinical algorithms, and future implications for larger implementation of this agent into everyday clinical practice.

## 1. Introduction

The P2Y12 receptor is one of the key pharmacological targets in preventing thrombotic phenomena in patients with coronary artery disease (CAD) and acute coronary syndromes (ACS), considering its key role in platelet aggregation. This receptor has a distinct role in platelet activation, as the interaction of ADP with P2Y12 amplifies the response to other pro-thrombotic stimuli and contributes to the increased thrombogenicity observed in injured arterial walls [1]. The potential effect of this receptor’s inhibition was understood early on via numerous preclinical and clinical studies, especially in the era of more potent agents. This has led to the everyday use of oral P2Y12 inhibitors, in addition to aspirin, in patients with ACS and patients undergoing percutaneous coronary interventions (PCI) [2]. More recently, studies have established the role of potent P2Y12 inhibitor monotherapy after PCI, which offers similar ischemic protection with less bleeding events, an outcome of interest particularly in patients considered of high bleeding risk [3,4]. Despite oral inhibitors being one of the cornerstones of post-ACS treatment, there are some limitations to their use in the acute setting. The 2023 European Society of Cardiology Guidelines on the Management of Acute Coronary Syndromes state that dual antiplatelet therapy (DAPT), with aspirin and an oral P2Y12 inhibitor, is the recommended default strategy for all patients presenting with ACS, particularly selecting prasugrel or ticagrelor over clopidogrel, and prasugrel over ticagrelor when the patient is expected to undergo PCI [5]. However, a subset of patients presenting with ACS would not be able to receive oral treatment, nor would patients presenting with cardiogenic shock (CS) and mechanical ventilation, among other instances.

In an aim to address the aforementioned limitations of oral P2Y12 inhibitors and offer a potential alternative for specific patient phenotypes during PCI, a parenteral inhibitor, cangrelor, was developed and evaluated for clinical use. Cangrelor is an intravenous direct, reversible P2Y12 inhibitor, which is characterized by a rapid onset and offset of platelet inhibition. Several studies have documented the safety and efficacy of cangrelor in patients undergoing PCI, mostly in comparison to clopidogrel [6]. However, guidelines do not offer specific indications for its use in the periprocedural setting. Namely, the ESC guidelines suggest the use of cangrelor in P2Y12 inhibitor-naïve patients and in an individualized manner, with the decision made based on each case’s characteristics, such as inability to receive oral treatment [5]. American guidelines support the use of cangrelor with a 2b recommendation in order to potentially reduce periprocedural ischemic events, stating, however, that no direct comparison with oral potent inhibitors is available [7]. Given the distinct pharmacological characteristics of this agent, compared to available oral alternatives, and the potential benefits of its use, identifying the ideal phenotypes treated with cangrelor could assist not only clinical practice but also future research efforts. Therefore, this review will summarize the pharmacological properties of P2Y12 inhibition and cangrelor, discuss the key clinical trials that support its use in the clinical setting, analyze specific indications for its use, and provide future perspectives on the development and use of this agent.

## 2. Pharmacology of Cangrelor

### 2.1. The Role of P2Y12 Receptor and Its Inhibitors in Thrombosis

The P2Y12 receptor is a G-protein-coupled receptor found in platelets, as well as other cells (microglia, immune), that binds and is activated by ADP and ATP. It is a member of the P2Y receptor family, which includes a total of eight receptor subtypes. When activated, these receptors initiate several intracellular pathways, including phospholipase C and adenylyl cyclase, which lead to tissue-dependent effects, i.e., in platelets, they result in platelet aggregation [8]. It is important to note that platelets have both P2Y12 and P2Y1, with activation of both receptors being necessary in order to induce a response. In more detail, when platelets interact with pro-thrombotic factors (thromboxane A2, von Willebrand factor, collagen, thrombin), platelet adhesion is initiated, whilst ADP is secreted from dense granules. The interaction of ADP with P2Y12 activates the receptor and further induces platelet aggregation as well as indirectly amplifies this response via enhancement of the positive feedback of other pro-aggregatory molecules or of the ADP-P2Y1 receptor interaction, which promotes platelet recruitment, dense granular secretion, and thrombus formation [9]. These effects result from the amplification of the PI3K pathway. However, evidence shows that P2Y12 signaling also inhibits cAMP formation, which could limit the anti-thrombotic effects of prostacyclin and adenosine-mediated signaling. Importantly, studies indicate that the P2Y12 receptor has an integral part in platelet aggregation in shear stress conditions when examining thrombus formation in flow conditions [10].

The pharmacological inhibition of the P2Y12 receptor started to be clinically performed even before researchers were able to clone the receptor with the administration of the thienopyridine molecules ticlopidine and clopidogrel [11]. Both agents are non-active prodrugs that become active when metabolized by the cytochrome P450 in the liver. They irreversibly bind to the P2Y12 receptor, resulting in its inactivation and, therefore, reduction of its mediated pro-thrombotic actions [11]. Several landmark studies established the safety and efficacy of clopidogrel administration in ACS, whilst this agent served as the comparator drug for more recent P2Y12 inhibitors, such as ticagrelor and prasugrel [12]. Given the abundance of evidence supporting their safety and efficacy, the use of oral P2Y12 inhibitors is one of the cornerstones of treatment in ACS [5], while post-procedural dual antiplatelet therapy (DAPT) with aspirin and an oral P2Y12 inhibitor is also key in order to avoid post-interventional thrombotic sequelae, particularly early on [2].

### 2.2. Cangrelor

Although the use of oral P2Y12 inhibitors is common practice in the management of ACS, they also have several limitations. To begin with, their onset of action is delayed, thereby leaving a significant periprocedural period uncovered by antiplatelet therapy. Second, oral administration in instances of systemic hypoperfusion due to cardiogenic shock may limit the bioavailability of oral agents, whilst, in advanced stages of shock, an inability to administer oral agents may be present. Considering these limitations, cangrelor, which is a nonthienopyridine analog, has been developed as the only intravenous, reversible inhibitor. Otherwise known as N-2-methylthio-ethyl-2-(3,3,3-trilflouroprpylthiol)-5′-adenyl acid, cangrelor selectively binds to the P2Y12 receptor reversibly, therefore inactivating the aforementioned pro-thrombotic cascade [13]. Compared to oral agents, cangrelor is reversible with significantly faster onset and platelet inhibition at a steady state. This agent has been developed from the ATP with some modifications (replacement of methylene groups with halogen, the addition of non-polar moieties and sulfide-like chain) that provide higher affinity with the P2Y12 receptor and higher resistance to ectonucleotidases. Similar to oral P2Y12 receptor inhibitors, cangrelor quickly (in a few minutes) binds to the P2Y12 receptor, blocking ADP-induced platelet activation and limiting other responses, including dense granule release and pro-thrombotic receptor expression [14]. However, investigations have hinted toward further antiplatelet effects of this molecule, on top of the blockage of the receptor, via a P2Y12-independent increase in cyclic AMP [15]. More specifically, cangrelor is able to increase intracellular cyclic AMP levels, an effect known to inhibit platelet aggregation, and do so independently of the P2Y12 signaling pathway via a separate G-protein-coupled route, therefore leading to enhanced antiplatelet effect. However, this mechanism is still under investigation, as other studies do not show similar evidence supporting an independent cyclic AMP increase [16]. In regard to bioavailability, cangrelor is mostly distributed intravascularly and does not have significant renal or hepatic metabolism, in contrast to oral P2Y12 inhibitors [17]. Finally, no sex-related differences have been found, with similar ischemic protection and no difference in the bleeding risk between males and females treated with cangrelor [18].

The first studies with the use of this agent were published in the early 2000s, when the drug was known as AR-C69931MX, by Storey et al. [19]. They included patients with unstable angina or non-q-wave MI who received the drug with stepped dose increments over 3 h to a plateau of either 2 mcg/kg/min for 21 h (n = 12) or 69 h (n = 13) or 4 mcg/kg/min for up to 69 h (n = 14). At the evaluation of patients at 24 h, the platelet activity was 96.0, 95.0, and 98.7% for each group, respectively, while at 1 h post-infusion, platelet activity was 36.2, 20.7 and 40.9%, respectively. Importantly, this study validated the safety and tolerability of the agent, as well as its short plasma half-life, which was under 9 min. Further studies [20] also showed almost complete platelet inhibition with a 4 mcg/kg/min continuous infusion, reaching its maximal effects at 15 min post-infusion and returning toward normal levels 15 min after infusion termination, with no significant increase in bleeding events or other drug-related adverse events. Regarding cangrelor bolus infusion, Akers et al. [13] compared a 15 mcg/kg bolus followed by a 2 mcg/kg/min infusion or a 30 mcg/kg bolus followed by a 4 mcg/kg/min infusion for 1 h in healthy individuals. Following bolus administration, the maximum platelet inhibition is achieved at 2 min and is maintained throughout the continuous infusion. Complete platelet recovery in both regimens was found after 60–90 min of infusion termination, with a half-life of 2–6 min. Moreover, despite the comparison of the two regimens having no significant difference, the authors note that the second regimen produced more potent and consistent platelet inhibition [13].

## 3. Clinical Evidence with the Use of Cangrelor

### 3.1. Landmark Randomized Studies

Having established the efficacy of cangrelor in platelet inhibition and understood its pharmacological properties with the aforementioned studies, investigators further aimed to evaluate the safety and efficacy of cangrelor in comparison to clopidogrel in three large randomized studies (CHAMPION trial program) (Table 1). In their study, Bhatt et al. [21] included (CHAMPION PLATFORM) 5362 patients undergoing PCI for ACS or CCS who were not pretreated with clopidogrel and randomized the group equally to either receive cangrelor or placebo at the time of the intervention, followed by 600 mg of clopidogrel. The primary endpoint of this trial was the composite of mortality, MI, or ischemia-driven revascularization at 48 h post-PCI. The trial, which had a superiority design, was terminated early due to its unlikely ability to meet the superiority margins. More specifically, the primary endpoint was not significantly different between the two arms, as it occurred in 7.0% (185/2654 patients) of the cangrelor arm and 8.0% (210/2641 patients) of the placebo arm (*p* = 0.17). However, the investigators reported significantly less stent thrombosis events and all-cause mortality at 48 h in the cangrelor cohort, as well as an increase in major bleedings (5.5 vs. 3.5%; *p* < 0.001), mostly due to more groin hematomas (*p* < 0.001). Interestingly, a sub-analysis of the trial [22] using the universal definition of MI, compared to the original definition used by the steering committee, showed reduced rates of the primary endpoint in both arms, which, however, still did not reach statistical significance (cangrelor 4.9% vs. placebo 6.0%; OR: 0.80; 95%CI: 0.63–1.02).

CHAMPION PCI [23] was a similar trial, enrolling 8877 patients undergoing PCI for CCS or ACS, in whom, however, cangrelor and clopidogrel 600 mg or placebo and clopidogrel 600 mg were pre-administered before the intervention. The primary endpoint of the trial was the same as the CHAMPION PLATFORM, while this trial was also stopped early due to prospected failure to reach statistical significance. The use of cangrelor resulted in non-superiority, compared to placebo, in regard to the primary endpoint at 48 h (7.5 vs. 7.1%; OR 1.05; 95%CI: 0.88–1.24) or 30-day follow-ups. Major bleeding events were not significantly different between groups; however, they were borderline significantly increased with cangrelor when considering the Acute Catheterization and Urgent Intervention Triage Strategy criteria (5.6 vs. 2.9%; *p* = 0.06). Stent thrombosis was also not significantly different between groups.

Aiming to better understand the effect of cangrelor in periprocedural ischemic events, the investigators of the two trials performed an analysis of the two cohorts, altering the definition of periprocedural MI in accordance with the universal definition of MI. In this context, despite no significant difference being found when calculating events using the original endpoint, when using the universal definition, the primary endpoint was significantly reduced in the cangrelor arm (OR 0.82, 95%CI 0.68–0.99, *p* = 0.037) [24]. Furthermore, stent thrombosis events were significantly reduced (0.2 vs. 0.4%; *p* = 0.018), while major bleeding events (according to the TIMI criteria) and blood transfusions were not different between groups.

Expanding the knowledge on the safety and efficacy of cangrelor, the CHAMPION PHOENIX [25] trial included 11,145 undergoing elective or urgent PCI, randomly assigned to either receive a bolus and a continuous cangrelor infusion or receive a loading dose of clopidogrel. The primary efficacy endpoint was a composite of death, MI, ischemia-driven revascularization, or stent thrombosis at 48 h. This trial met its primary endpoint, showing a significant reduction of the composite of events at 48 h in those receiving cangrelor (4.7 vs. 5.9%; *p* = 0.005). Furthermore, the trialists reported a significant reduction in stent thrombosis events (0.8 vs. 1.4%; *p* = 0.01), with no significant difference in major bleeding events. In sub-analyses of the trial, it has been shown that cangrelor is equally safe and effective, regardless of patient presentation (stable angina or ACS), with similar protection from ischemic events and stent thrombosis along with no difference in bleeding events between the two groups [26]. Similar results have also been reported in the comparison of male versus female patients, with cangrelor showing comparable efficacy in preventing cardiovascular events and stent thrombosis in both genders [27]. Specifically, despite more bleeding events being present in women, the net clinical benefit remained in favor of cangrelor, as it led to a greater reduction in ischemic endpoints than in men (stent thrombosis reduction of 61% in women vs. 16% in men). Importantly, the protective effect of cangrelor, compared to bolus clopidogrel, remained in patients with concurrent bivalirudin treatment, as White et al. [28] indicated. Finally, Genereux et al. [29] reported that, despite generally low (0.8% of the overall cohort), periprocedural stent thrombosis was significantly associated with adverse cardiovascular events at follow-up, while it was significantly reduced with the use of cangrelor; thus, reducing the events of this adverse event predictor markedly improved the outcome of patients under cangrelor treatment.

Collectively summarizing available randomized data, a patient-level analysis of all three CHAMPION trials was performed by Steg et al. [30], including 24,910 patients. Of note, only 11.6% of the population presented with STEMI, while 57.4% had an NSTEMI diagnosis. The primary efficacy endpoint was a composite of mortality, MI, ischemia-driven revascularisation, or stent thrombosis at 48 h. The use of cangrelor significantly reduced the odds of the primary outcome by 19% (3.8 vs. 4.7%; *p* = 0.0007), as well as the stent thrombosis rate by 41% (0.6 vs. 0.8%). When excluding stent thrombosis from the primary endpoint, cangrelor use also resulted in a significant reduction of the composite endpoint by 19%. These benefits of cangrelor remained significant at 30-day follow-ups. Regarding safety, no significant difference was present in severe or moderate bleeding events or need for transfusion between groups, except a significant increase in mild bleeding in the cangrelor arm (16.8% vs. 13.0%, *p* < 0.0001). The results of the aforementioned studies and this analysis confirmed the safety and efficacy of cangrelor, particularly in reducing periprocedural stent thrombosis, and therefore led to the approval of the agent by the regulatory authorities in 2017.

**Table 1:** Major randomized trials on the use of Cangrelor (CHAMPION trial series).

Trial Name	Number of Participants	Setting	STEMI Presentation (%)	Cangrelor Infusion Characteristics	Post-PCI Treatment	Primary Endpoint	Stent Thrombosis	Bleeding Events
CHAMPION-PLATFORM [21]	5362	PCI for ACS or CCS	Excluded	30 mcg/kg bolus and 4 mcg/kg/min infusion for ≥2 h or until the end of the procedure	Clopidogrel 600 mg in both groups	No difference in the composite of mortality, IDR, or Mi at 48 h (7.0 vs. 8.0%; *p* = 0.17)	Significantly reduced thrombotic events in the cangrelor arm at 48 h (0.2 vs. 0.6%; *p* = 0.02)	No significant difference in blood transfusions Significant increase in major bleedings (5.5 vs. 3.5%; *p* < 0.001)
CHAMPION-PCI [23]	8877	PCI for ACS or CCS	11%	30 mcg/kg bolus and 4 mcg/kg/min infusion	Clopidogrel 600 mg in both groups	No difference in the composite of mortality, IDR, or Mi at 48 h (7.5 vs. 7.1%; *p* = 0.59)	No significant differences between arms (0.2 vs. 0.3%; *p* = 0.34)	Major bleeding according to the ACUITY criteria: higher with cangrelor, approaching statistical significance (3.6% vs. 2.9%; *p* = 0.06) No difference in major bleeding according to the TIMI criteria or according to GUSTO criteria.
CHAMPION-PHOENIX [25]	11,145	PCI for ACS or CCS	17.6%	30 mcg/kg bolus and 4 mcg/kg/min infusion	Clopidogrel 600 mg in the cangrelor group, placebo in the control arm	Significant reduction in the composite of mortality, IDR, or MI at 48 h (4.7 vs. 5.9%; *p* = 0.01)	Significant reduction of stent thrombotic events at 48 h (0.8 vs. 1.4%; *p* = 0.01)	No significant difference in major bleeding events (both 0.1%) Trend toward increase in minor bleedings with cangrelor (0.2 vs. 0.1%; *p* = 0.08)
Pooled CHAMPION trials analysis [30]	24,910	PCI for ACS or CCS	11.3%	30 mcg/kg bolus and 4 mcg/kg/min infusion	As per trial	Significant reduction of the composite death, MI IDR, or stent thrombosis at 48 h (3.8 vs. 4.7%; *p* = 0.0007)	Significant reduction in stent thrombosis events between groups (0.5 vs. 0.8%; *p* = 0.0008)	No difference in GUSTO moderate bleedings (0.6 vs. 0.4%), but increase in GUSTO mild bleedings (16.8 vs. 13.0%; *p* < 0.0001)

Abbreviations: PCI: percutaneous coronary intervention; ACS: acute coronary syndrome; CCS: chronic coronary syndrome; STEMI: ST Elevation Myocardial Infarction; IDR: ischemia-driven revascularization; MI: myocardial infarction; TIMI: Thrombolysis in Myocardial Infarction; GUSTO: Global Use of Strategies to Open Occluded Coronary Arteries; ACUITY: Acute Catheterization and Urgent Intervention Triage Strategy.

### 3.2. Real-World Use of Cangrelor

On the basis of randomized trials, a limited number of real-world registries aimed to report the actual use of cangrelor in PCI centers. Available studies mostly report the use of cangrelor in patients with STEMI or out-of-hospital cardiac arrest, with only a small subset of patients undergoing an elective PCI. In more detail, the analysis of the Swedish Coronary Angiography and Angioplasty Registry [31] included 899 STEMI patients treated with cangrelor, who were compared to 4616 patients not receiving cangrelor. Another 16 non-STEMI patients were treated with cangrelor throughout the registry, who, however, were excluded. In all of the participating hospitals, the use of cangrelor significantly varied (4–36%), while in 19% of patients, the use of cangrelor was in STEMI-cardiac arrest patients. Prehospital treatment with ticagrelor was given in more than half of instances of cangrelor use, while those receiving the intravenous agent were more frequently high-risk patients (i.e., left main PCI, use of thrombus aspiration devices, and cardiac arrest). Overall, concurrent pre-PCI loading with ticagrelor was relatively common (33.4 vs. 70.6% in the cangrelor and control arm, respectively). While noting the increased risk phenotype of patients receiving cangrelor and the absence of comparison between the groups, 30-day all-cause morality was numerically higher in the cangrelor arm (15.1 vs. 6.0%), with the rates being reduced in an analysis excluding cardiac arrest and rescue PCI (8.8 vs. 5.3%). Importantly, the stent thrombosis rate was similar between the cangrelor (0.7%) and clopidogrel (0.8%) groups.

Similar results were reported by Thim et al. [32], including 991 patients treated with cangrelor. A total of 87.7% had an acute procedure, with 73% of patients presenting with STEMI, 8% with NSTEMI, and 10.8% with out-of-hospital cardiac arrest. Importantly, 95.9% of patients treated with cangrelor did not receive P2Y12i pretreatment. Regarding outcomes, all-cause death at 48 h was 2.6% (1.8% cardiac death), while the rates of stent thrombosis and MI remained low (0.2% for both, respectively). Therefore, the real-world analyses show that cangrelor is typically used in ACS and high-risk individuals, mostly in cases when P2Y12 inhibitor pretreatment is not feasible or when the rapid, potent initiation of platelet inhibition could be considered of benefit to the patient. Furthermore, it is evident that, despite not being tested in a randomized setting, concurrent ticagrelor and cangrelor treatment could regard up to a third of cangrelor-treated patients in clinical practice.

In regard to the efficacy of cangrelor, more recently, the INVEST-STEMI real-world study compared patients with STEMI receiving either cangrelor (n = 312) or tirofiban (n = 315) at the time of primary PCI (pPCI) [33]. The primary efficacy endpoint was the angiographic evidence of thrombolysis in MI (TIMI) flow < 3 after pPCI, while the primary safety outcome was the in-hospital occurrence of BARC (Bleeding Academic Research Consortium) 2–5 bleedings. Patients receiving cangrelor had lower ischemia time, while the risk for TIMI flow < 3 was significantly lower in the cangrelor group (adjusted OR: 0.40; 95% CI: 0.30–0.53). Importantly, the bleeding risk was comparable between the two arms (adjusted OR:1.35; 95% CI: 0.92–1.98). Therefore, this study shows that compared to other intravenous agents, the use of cangrelor is associated with improved myocardial perfusion without increasing clinically relevant bleedings. However, further data, in comparison with other common intravenous agents as well as potent oral P2Y12 inhibitors, are necessary in order to establish the benefit of cangrelor in pPCI patients.

### 3.3. Cangrelor-Oral P2Y12i Co-Administration and Transition

A recognized advantage of cangrelor is its rapid onset and offset of action, which could benefit PCI patients in the timeframe between oral P2Y12 inhibitor administration and platelet inhibition onset. Initial efforts were made toward bridging cangrelor with clopidogrel in order to provide post-procedural antiplatelet protection. In this context, Steinhubl et al. [34] investigated whether concomitant cangrelor with clopidogrel loading dose versus clopidogrel following cangrelor infusion has any difference in platelet inhibition in healthy individuals. The investigators reported that, when examined in isolation, cangrelor and clopidogrel both achieved the expected levels of platelet inhibition. However, when cangrelor and clopidogrel are co-administered, the anticipated platelet inhibition does not occur, in contrast to the clopidogrel after cangrelor infusion strategy. This observation can be explained by the fact that cangrelor strongly binds with the P2Y12 receptor, thus potentially preventing clopidogrel from acting. Given the short time the active metabolite of clopidogrel remains in circulation (approximately 20 min), it is logical to assume that co-administration of these two regimens does not result in sufficient platelet inhibition. Similar results were found by Dovlatova et al. [35], reporting an interaction between cangrelor and clopidogrel/prasugrel metabolites. Specifically, the study showed that preincubation of blood with cangrelor prior to the addition of the active metabolites of clopidogrel or prasugrel reduced their efficiency in irreversibly antagonizing P2Y12 receptor; however, their effects were maintained when they were administered prior to cangrelor use. Considering this fact, the use of the more potent P2Y12 inhibitor ticagrelor, which remains in circulation for a longer time, was explored to bridge the use of cangrelor.

As discussed, from the time of administration, ticagrelor exhibits its maximum antiplatelet effects after 2–4 h [36]. Therefore, concurrent intravenous and oral P2Y12 inhibition could account for the latency of oral inhibition, providing sufficient antiplatelet coverage throughout the intraprocedural and postprocedural periods. However, co-administration of these agents could lead to significant drug–drug interaction, especially considering that this hypothesis was not tested in the CHAMPION trials. Given the aforementioned increased ticagrelor and cangrelor co-administration in the real world, investigators aimed to investigate whether co-administration, as well as bridging of the two agents, is safe and feasible.

Regarding co-administration of cangrelor with ticagrelor, Franchi et al. [37] included 50 patients in the CANTIC study who were either randomized to receive cangrelor infusion (2–4 h) or placebo. All patients had received a loading ticagrelor dose (180 mg) prior to pPCI. The study evaluated platelet reactivity in both cohorts and showed that cangrelor use was associated with reduced P2Y12 reaction units at 5 min post-infusion, which remained significantly reduced throughout the infusion period (*p* < 0.001). Importantly, platelet reactivity was significantly reduced in patients with high on-treatment platelet reactivity, while after infusion discontinuation, no evidence of drug–drug interaction was found. Therefore, this study showed that the co-administration of cangrelor in patients treated with ticagrelor is safe and effective, with no evidence of adverse events related to their interaction.

In a similar context, the SWAP-5 study [38] randomized 20 patients with CAD undergoing PCI to either receive cangrelor bolus and infusion or placebo one hour after the administration of a loading dose of ticagrelor. As shown in the CANTIC study, the addition of cangrelor, in comparison to placebo, resulted in significantly reduced P2Y12 reactivity units at both 30 min and 1 h after starting the infusion. Moreover, at 2 h after cangrelor termination, both groups had similar levels of P2Y12 reactivity units as well as no difference in the plasma levels of ticagrelor, confirming the non-inferiority of the combined approach to isolated ticagrelor use. Thus, cangrelor enhances the antiplatelet effect during the procedure, with no pharmacokinetic or pharmacodynamic difference present after discontinuing the agent.

Comparing the use of cangrelor in patients pretreated with any oral P2Y12 inhibitor, the CAMEO registry [39] included 385 patients having received cangrelor infusion and a P2Y12 inhibitor no more than 24 h prior to the procedure in order to assess the safety of this strategy in respect to bleeding events. No significant difference in bleeding rates was found among cangrelor-treated patients with and without oral P2Y12 inhibitor preexposure (6.5% vs. 8.8%; adjusted OR: 0.62; 95% CI, 0.38–1.01), as well as in the comparison between pretreatment with high and low potency P2Y12 inhibitors. Moreover, bleeding events were observed in 5.0%, 10.7%, and 3.2% of patients treated with cangrelor <1, 1 to 3, and >3 h after the last oral P2Y12 inhibitor dose, with no significant differences between groups.

De Luca et al. [40], in the ARCANGELO study, investigated, besides the safety of cangrelor, the effect of transitioning to oral P2Y12 inhibitors. Therefore, they included 1004 ACS patients undergoing PCI, with 60% presenting with STEMI. All patients received cangrelor bolus and a subsequent continuous infusion. Regarding the transition to P2Y12 treatment, 73.4% of patients received ticagrelor, 12.8% prasugrel, and 13.9% clopidogrel. Overall, 30-day bleeding, according to the BARC criteria, occurred in 5.2% of patients, with the majority (4.7%) being mild. Most bleeding events occurred with ticagrelor, without, however, providing a statistical comparison between different oral P2Y12 inhibitors, while only in five patients did the investigators link the bleeding with the use of cangrelor. Major adverse cardiac events occurred in 1.4% of patients. This study, therefore, shows the low rates of bleeding and MACE when switching from cangrelor to oral inhibitors.

Finally, SWAP 6 expanded the evidence on the transition from cangrelor to prasugrel in PCI patients [41]. They included 77 patients randomized to either receive prasugrel only at the start of PCI, receive cangrelor plus prasugrel concomitantly at the start of PCI, or receive cangrelor at the start of PCI plus prasugrel at the end of infusion. The primary endpoint was non-inferior in platelet reactivity, as measured with P2Y12 reaction units, at 4 h after randomization between the concomitant cangrelor plus prasugrel vs. prasugrel-only groups. At 4 h, P2Y12 reaction unit levels were significantly lower with prasugrel only compared to combined cangrelor and prasugrel; therefore, the combined strategy failed to meet the prespecified non-inferiority margin and, thus, suggested a potential drug–drug interaction between those agents. However, this finding needs to be further explored in future trials in order to be better understood and validated.

## 4. Cangrelor and Microcirculation/MVO

### 4.1. P2Y12 Blockade and Microcirculation

Recent advancements in the field of coronary physiology have allowed us to better understand the impact of the status of the coronary microcirculation post-PCI in patients presenting with ACS. There are several pathophysiological mechanisms contributing to microcirculatory dysfunction in the post-ACS time period, including endothelial dysfunction, inflammation, and microvascular obstruction (MVO) [42,43]. Moreover, along with the aforementioned parameters and their role on infarct size, reactive oxygen species have also been related to infarct size, while amelioration of oxidative stress has been shown to reduce infarct extent [44,45,46]. Notably, studies have shown that despite primary PCI revascularizing the vast majority of epicardial stenoses, the rate of complete myocardial revascularization, considering the microcirculation, is not much higher than 50% [47]. Typical indices used for microcirculation assessment post-ACS include, as in stable disease, coronary flow reserve (CFR) and the index of microvascular resistance (IMR). Meta-analysis data show that in patients with ACS, abnormal CFR is associated with a 3.76-fold increase in MACE [48], as well as increased rates of left ventricular diastolic dysfunction and adverse functional left ventricular remodeling [49]. In regard to IMR, which is more commonly used given that its results are independent of the epicardial artery disease and, therefore, better indicate the isolated coronary macrovasculature physiology [50], a value greater than 40 is significantly indicative of increased MACE [51,52,53]. Moreover, IMR has been related to the extent of the infarct size post-PCI, with IMR > 40 being significantly associated with increased infarct size, as well as increased presence of intramyocardial hemorrhage and MVO [54]. Other studies, notably, highlight that despite IMR being normal in 1/3 of patients with MVO diagnosed by cardiac magnetic resonance, patients with both a diagnosis of MVO and increased IMR were at the highest risk for mortality and adverse events [55]. Novel indices, such as microvascular resistance reserve (MRR), which have the potential to overcome the limitations posed by both CFR and IMR [56], have also been recently tested in the context of ACS, showing that decreased MRR is predictive of adverse cardiovascular outcomes [57], with future studies aiming to further establish this indice, as well as evaluate its relationship with MVO. Considering the above, the role of microvascular dysfunction is crucial in the post-ACS period, while interventions improving microvasculature physiology could enhance patient outcomes and prevent myocardial damage or adverse remodeling.

Considering the broad use of oral P2Y12 inhibitors in patients after PCI, several investigators have evaluated if they exert any beneficial properties to coronary microcirculation or could ameliorate MVO. Studies with ticagrelor in both patients with ACS and stable disease have shown that it can improve coronary microcirculation indices [58,59]. Similar results have also been shown for clopidogrel. However, in comparative analyses, ticagrelor outperforms the use of clopidogrel. Specifically, Park et al. [60] have reported that in patients receiving a loading dose of clopidogrel versus ticagrelor, IMR immediately after PCI was significantly lower in the ticagrelor compared to the clopidogrel group (22.2 ± 18.0 vs. 34.4 ± 18.8 U, *p* = 0.005); however, infarct size was not different at 3-month follow-ups. Notably, the beneficial effects in microcirculation and related indices are still present at 6-month follow-ups, as investigators [61] have demonstrated significantly lower IMR (15.57 ± 5.65 vs. 21.15 ± 8.39; *p* < 0.01) and higher CFR (3.85 ± 0.72 vs. 3.37 ± 0.76; *p* < 0.01), with no difference in fractional flow reserve in patients under ticagrelor versus clopidogrel treatment. Similarly, data from updated meta-analyses suggest that ticagrelor use results in greater reduction of IMR (weighted mean difference: −6.23, 95% CI: −8.41 to −4.04) and greater increase in CFR (weighted mean difference: 0.38; 95% CI: 0.18 to 0.57) [62], as well as improved corrected TIMI frame count and myocardial perfusion, as assessed by myocardial blush grade [63]. Pathophysiological theories for these benefits include the more potent platelet inhibition associated with ticagrelor, which could limit the formation of microthrombi and the subsequently increased microvascular resistance due to MVO. This hypothesis is confirmed by a recent study, which showcased the dependency of IMR reduction on the percentage of platelet inhibition [61]. Moreover, this theory is also supported by the fact that in most studies, prasugrel use results in similar benefits to ticagrelor [64]. Other working theories include the adenosine theory, referring to the increase in adenosine after ticagrelor administration due to the blockage of the equilibrative nucleoside transporter-1 receptor, which results in greater local extracellular adenosine level, especially on sites of increased thrombus formation, such as ischemic tissues [65]. However, this theory is conflicted by investigations showing no substantial increase in adenosine and, as aforementioned, similar effects of ticagrelor and prasugrel in microvascular injury post-STEMI [64].

Finally, the role of P2Y12 inhibitors has also been evaluated in regard to endothelial dysfunction, which is a key pathogenetic factor for CMD [66]. Preclinical studies have demonstrated that administration of P2Y12 inhibitors attenuates endothelial dysfunction via reducing the expression of inflammatory molecules (including vascular cell adhesion molecule-1), macrophage accumulation, and lipid deposition whilst ameliorating the impairment of endothelium-dependent vasodilation by adenosine diphosphate via decreased phosphorylation of the JNK [67]. Further investigations have shown that treatment with P2Y12 inhibitors or attenuation of the P2Y12 receptor in knockout models also leads to reduction of interleukin-6 and tumor necrosis factor-a [68,69], inhibition of platelet–leukocyte interaction and platelet p-selectin expression [70], which are all factors contributing to vascular damage and endothelial dysfunction. Moreover, these agents also show antioxidant properties, as studies [71,72] have reported a protective effect of ticagrelor against endothelial dysfunction by restoration of endothelial nitric oxide synthase activity and reduction of reactive oxygen species generation, mitochondrial dysfunction, and endoplasmic reticulum stress. Considering the favorable preclinical data, clinical studies have also shown benefits in some studies [73,74] in regard to floe-mediated dilation (FMD); however, other investigations did not report any benefit [75,76]. In this context, a recent meta-analysis [77], including 21 studies, reported that ticagrelor resulted in a significant increase in FMD (weighted mean difference: 1.48; 95% CI: 0.36, 2.60), reactive hyperemia index (weighted mean difference: 0.06; 95% CI: 0.00, 0.13), and circulating progenitor endothelial cells (weighted mean difference: 13.84; 95% CI: 5.70, 21.98), therefore showing a potential benefit in endothelial dysfunction, but noting the high heterogeneity of studies, prompting further investigation.

### 4.2. The Role of Cangrelor

In parallel with the investigations employing oral P2Y12 inhibitors, cangrelor has been studied in both preclinical and clinical settings regarding its effect on infarct size (Table 2). To this date, there are limited available studies reporting changes in CFR or IMR. However, considering the association of infarct size with MVO, increased microvascular resistance, and microvascular dysfunction, these studies provide the first evidence of a potential effect of this agent in the microvasculature.

Studies on animal models have showcased the benefit of cangrelor on infarct size. Yang et al. [78] showed in a rabbit heart model that cangrelor administration reduced infarct size from 38% of the ischemic zone in control hearts to only 19% in a dose-dependent manner and in a similar extent to ischemic postconditioning. However, its protective effects were lost if cangrelor was infused 10 min after revascularization. Similar results have been noted in monkey hearts, where effective platelet aggregation blockage (94%) was also highlighted [79]. However, there is still a gap in knowledge regarding the pathophysiological mechanism behind these effects, as investigators note that they may not be related to direct antiplatelet effects rather than reperfusion injury protection. On that note, Cohen et al. [80] showed that the protective effects of cangrelor on the infarct size are exerted even in animal models with thrombocytopenia, while sphingosine kinase, a protein associated with the protective mechanism of ischemic postconditioning, was found to be important for the regulation of these effects.

On the contrary, when considering clinical trials, the results from the use of cangrelor are neutral. Ubaid et al. [81] evaluated the effect of cangrelor in a randomized setting on myocardial microvascular function and infarct size. They enrolled 100 patients with STEMI, who were assigned 1:1 to receive either cangrelor or ticagrelor, and compared the effect of these agents regarding infarct size and microcirculation parameters between the groups. Although P2Y12 inhibition at the time of first balloon inflation was significantly greater with the use of cangrelor, with no difference in mean platelet reactivity at 4 and 24 to 36 h, IMR, and final infarct size were similar between groups. Similar results were reported in the PITRI study by Bulluck et al. [82], showing no significant difference in acute MI size or incidence and extent of MVO despite enhanced platelet reactivity reduction with the use of cangrelor. However, as the authors note, a relatively low percentage of patients with high residual platelet reactivity at baseline (43%), as well as an increased chest pain onset balloon time in the cangrelor group, may have ameliorated the cardioprotective effects of cangrelor. Therefore, these studies do not currently support the effect of cangrelor on microcirculation, in contrast to the benefit observed in preclinical settings. Considering the number of limitations and the reduced number of available studies, it is evident that no safe conclusion can be extracted from these studies. Ongoing trials, such as the MIRACOR study (NCT06089577), which is estimated to be completed within 2026, will provide more clarity into the effect of cangrelor in microcirculatory indices and microvascular homeostasis.

**Table 2:** Preclinical and clinical studies evaluating the effect of cangrelor in microcirculation and infarct size.

Study Name	Study Type	Number of Participants	Intervention	Infarct Size Outcomes	MVO Outcomes	Microcirculation Outcomes
a. Preclinical/Animal Studies						
Yang et al. [78]	Preclinical	1 rabbit	Cangrelor infusion before an I/R injury	Reduction of the infarction area from 38% in an ischemic zone control to 19% in a dose-dependent manner	NR	NR
Yang et al. [79]	Preclinical	31 monkeys	Cangrelor infusion before an I/R injury	Regression analysis showed that infarct size in hearts treated with cangrelor (r = 0.93) is significantly different from the regression for control hearts (*p* < 0.001)	NR	NR
b. Clinical Studies						
Ubaid et al. [81]	Clinical	100 patients with STEMI	Cangrelor infusion or oral ticagrelor	No difference in infarct size (% of left ventricular mass) between groups (13.7 vs. 10.9%; *p* = 0.61)	NR	IMR was similar in both groups (30 vs. 28; (cangrelor 30; *p* = 0.52). No difference in the proportion of patients with IMR > 40 (40 vs. 24%; *p* = 0.11) No difference in CFR (1.3 vs. 1.4; *p* = 0.30)
Bulluck et al. [82]	Clinical	164 patients with STEMI undergoing CMR	Cangrelor infusion during primary PCI	No significant difference in acute MI size (% of left ventricular mass) (16.3 vs. 14.9%; *p* = 0.40)	No difference in the incidence of MVO between arms (47 vs. 48%; *p* = 0.99) No difference in the extent based on % of left ventricular (1.18 vs. 1.63%;) *p* = 0.46)	NR

Abbreviations: I/R: Ischemia/Reperfusion; IMR: Index of Microvascular Resistance; CFR: Coronary Flow Reserve; MI: myocardial infarction; STEMI: ST Elevation Myocardial Infarction; MVO: microvascular obstruction; CMR: cardiac magnetic resonance; PCI: percutaneous coronary intervention; NR: not reported.

## 5. Role in Cardiogenic Shock, Cardiac Arrest and Cardiac Surgery

### 5.1. Cardiogenic Shock/Cardiac Arrest

As stated by the 2023 European Guidelines for the Management of ACS [5], the role of cangrelor in the periprocedural setting may be even more enhanced in patients not able to receive oral antiplatelet agents, such as patients with cardiac arrest or patients with cardiogenic shock, in whom gastrointestinal hypoperfusion could limit the absorption of oral agents. Notably, as aforementioned, in real-world registries, the use of cangrelor due to cardiac arrest or cardiogenic shock is one of the key reported indications, compared to randomized trials that did not commonly include cardiogenic shock patients. Therefore, several studies have aimed to evaluate whether, in such patients with more severe presentation, the use of cangrelor infusion is safe and feasible and results in a comparable or better efficacy than oral counterparts.

Droppa et al. [83] evaluated 88 patients with cardiogenic shock treated with cangrelor and compared them with 88 matched patients from the IABP-SHOCK II trial regarding mortality at 30-day and 1-year follow-ups. Despite 30-day mortality not being different between groups (29.5 vs. 36.4%; *p* = 0.34), there was a non-significant trend toward reduced all-cause mortality with the use of cangrelor at 1 year (34.1 vs. 47.1%; *p* = 0.08). No difference was also found in the rates of in-stent restenosis (2.3% in both arms) or bleeding events (21.6 vs. 19.3%; *p* = 0.71); however, cangrelor administration was significantly associated with increased rates of TIMI flow grade improvement ≥ 1 during PCI (92.9 vs. 81.2%; *p* = 0.02)

Rymer et al. [84] also performed a sub-analysis of the CAMEO registry, including only patients presenting with cardiogenic shock. The total cohort comprised 249 patients, out of which 59% also had mechanical circulatory support. Patients with cardiogenic shock received longer infusions compared to the total number of patients in the registry (3.9 vs. 2 h), while lung disease and the presence of mechanical circulatory support were predictive of longer infusions. Notably, 24.1% of the patients had a bleeding event, and 41.8% experienced an adverse event, while a longer cangrelor infusion duration was significantly associated with an increased risk of bleeding. Similar results in this patient cohort were also reported by Katz et al., showing a bleeding event rate of 59%, with the majority of them being related to the cannulation site [85].

Moving further, Kordis et al. [86] evaluated the safety and efficacy of cangrelor in comatose patients due to out-of-hospital cardiac arrest undergoing PCI. A total of 30 patients were randomized in a 1:1 fashion, receiving either cangrelor infusion or placebo, and compared for the primary efficacy endpoint of platelet inhibition at 1, 3, 5, and 8 h as well as the primary safety endpoint of BARC 2,3 or 5 bleedings. All patients received ticagrelor via nasogastric tube upon intensive care unit admission. No significant differences were noted between the two arms regarding procedural success (TIMI flow), in-hospital survival, or BARC 2,3 or 5 bleedings. In regard to platelet reactivity, it was significantly decreased in those receiving cangrelor at 1 h (30 ± 45 vs. 221 ± 45 PRU; *p* < 0.001) and 3 h (24 ± 36 vs. 180 ± 67 PRU; *p* < 0.001), with no differences being noted at 5 and 8 h. Considering the limitation of the small study size (30 patients), this study showcased the profound platelet activity reduction in parallel with the absence of drug–drug interaction with ticagrelor, making cangrelor a potentially safe option for such patient phenotypes.

Zeymer et al. [87] included in their study patients with cardiac arrest due to myocardial infarction, with or without cardiogenic shock. A total of 303 patients were originally evaluated, with the primary outcome of stent thrombosis and recurrent MI (first 48 h) being reported in 2 patients (0.7%), while BARC 2–3 bleeding events in 11.2% BARC 5 events in 3.3% of the patients. Total in-hospital mortality was 41.6%, with cardiac mortality being 21.8%. In an analysis comparing 118 matched patients with the results of the CULPRIT-SHOCK trial [88], the investigators reported comparable TIMI 3 flow rate of the infarct artery between the two studies (91.5 vs. 87.9%), while patients treated with cangrelor had numerically fewer ischemic events (38.1 vs. 49.1%; *p* = 0.09 for the combined endpoint of cardiovascular death, stent thrombosis, re-myocardial infarction, stroke, and BARC 2–5 bleeding) and a trend toward less cardiovascular death (25.4 vs. 36.8%; *p* = 0.06). Therefore, this study highlights the overall low number of thrombotic events in cardiogenic shock patients undergoing PCI and receiving cangrelor treatment, which may be lower compared to oral P2Y12 treatment.

Aiming to synthesize the available studies, a meta-analysis by Ursoleo et al. [89] included 10 studies and 678 patients with cardiogenic shock, out of whom 26% needed mechanical circulatory support. Notably, the dose of cangrelor differed between studies, with 57% of patients receiving 4 mcg/kg/min, 37% 0.75 mcg/kg/min, and the rest less than 0.75 mcg/kg/min. The pooled hospital survival rate of this patient cohort was 66% (95%CI: 59–73%). Regarding safety, the pooled rate of major bleeding events was 17% (95%CI: 11–23%), while the pooled rate of stent thrombosis was 1% (95%CI: 0.3–2.3%), and of any thrombosis was 3% (95%CI: 0.4–7%). Therefore, these data highlight that cangrelor infusion is safe in patients with cardiogenic shock despite the frequent bleeding complications whilst highlighting that the optimal dosing of the continuous infusion has yet to be determined. In an aim to answer the aforementioned gaps in knowledge, the DAPT-SHOCK-AMI trial [90] is ongoing, randomizing patients to receive either intravenous cangrelor or crushed ticagrelor, with a primary composite endpoint of all-cause death, MI, or stroke at 30-day follow-ups. The completion of this study is anticipated to happen before the end of 2025 and will provide further guidance on the use of cangrelor in this special patient phenotype.

### 5.2. Cardiac Surgery

The role of cangrelor as a bridge for emergent cardiothoracic surgery and, most importantly, coronary artery bypass surgery (CABG) was recognized early, given the immediate offset of action of this agent, compared with oral P2Y12 inhibitors. The landmark study for this indication was the BRIDGE study [91], which randomized patients to either receive cangrelor or placebo infusion for at least 48 h after thienopyridine interruption, which was continued for 1–6 h after cardiac surgery. The primary efficacy endpoint was platelet reactivity, while the main safety endpoint was coronary artery bypass graft (CABG)-related bleeding. Regarding platelet activity, patients under cangrelor treatment had significantly higher rates of P2Y12 reactivity units below 240 (98.8 vs. 19.0%; *p* <0.001), while there was no evidence of a significant difference in CABG-related bleeding events between the two arms (11.8 vs. 10.4%; *p* = 0.763). Similar results have been reported in other cardiac surgery, real-world series, evaluating patients with implanted stents and supporting bridging with cangrelor [92], as well as in case series of emergency CABG, where cangrelor infusion allowed timely reversal of the antiplatelet effect without delays related to the longer offset of oral antiplatelet agents [93]. Considering these results, as well as results in non-cardiac surgery [94,95], the use of cangrelor is increasing in elective cases at high risk, with recent PCI and under DAPT treatment in order to provide sufficient antiplatelet coverage in the periprocedural period. However, there is a need for further trials evaluating the use of cangrelor in ACS patients undergoing emergent CABG, as it could provide a safer than oral antiplatelet options due to its immediate offset of action.

## 6. Clinical Practice Perspective

**Figure 1:**
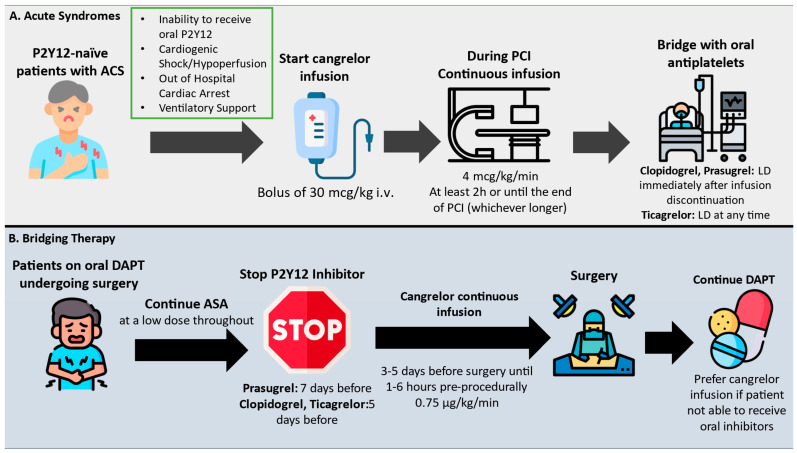
Algorithmic approach to the use of cangrelor in everyday practice. Abbreviations: ACS: acute coronary syndrome; ASA: aspirin; DAPT: dual antiplatelet therapy; LD: loading dose; PCI: percutaneous coronary intervention.

The use of cangrelor in everyday clinical practice is increasing, especially in high-risk P2Y12 naïve patients presenting with ACS complicated by cardiogenic shock, high thrombotic burden, or cardiac arrest (Figure 1). Currently, as aforementioned, the ESC guidelines’ indication for the use of cangrelor is IIb in the aforementioned patient categories, considering the benefits derived from the CHAMPION trials. However, cangrelor could exert further benefit in real-world settings. When surgery is needed early after an ACS due to procedural complications, bleeding events, or mechanical complications of the MI, or there is a need for CABG, oral agents should have to be stopped longer in order to allow for platelet recovery (at least 48 h; however. full recovery could take up to 7 days). Therefore, in such cases, the use of cangrelor could allow for timely interventions. Moreover, considering the faster onset of action of oral inhibitors, at the time of the PCI, the patient may not have sufficient platelet inhibition periprocedurally. Therefore, in patients with increased platelet activity despite oral P2Y12 treatment, cangrelor could offer a solution toward achieving optimal outcomes. Of course, in patients with systemic hypoperfusion and cardiogenic shock, as described already, the benefit of parenterally delivering an antiplatelet agent versus selecting a compromised oral route that could alter bioavailability is apparent, and therefore cangrelor should be considered early in such instances.

Besides traditional indications, novel studies could provide further evidence for the use of cangrelor during PCI in order to prevent adverse events due to either a more sufficient antiplatelet reactivity reduction or pleiotropic effects. As it is well known, most preventive agents for post-ACS reperfusion injury have not performed well in clinical studies, similar to the effect of cangrelor in infarct size, microvascular function, and MVO. However, the positive preclinical data of both oral P2Y12 and cangrelor benefit in endothelial function and microcirculation suggest that there could be an effect of these agents in the clinical setting [78,79,80,81], though perhaps in distinct clinical phenotypes. Ongoing trials, as mentioned (NCT06089577), are going to evaluate whether there is any benefit of cangrelor use in coronary microvascular dysfunction and reperfusion injury. Other phenotypes that could be of benefit to investigate include high bleeding risk patients, where early reports show a potential increase in bleeding events in those fulfilling such criteria [96], and even an increase in both ischemic and bleeding events in the cangrelor, compared to the non-cangrelor group [97]. Considering these results, further evidence is needed in the high-bleeding-risk population to support the use of cangrelor infusion. Addressing prior studies’ limitations and focusing on better-selected patients is of the essence in order to understand more regarding any further benefit of cangrelor use in diverse clinical scenarios.

## 7. Conclusions

The use of cangrelor is a safe and efficient alternative to oral P2Y12 inhibitors, with a potent and fast antiplatelet effect and no drug–drug interaction with oral agents, which may be particularly beneficial in reducing platelet activity early on during PCI. Beyond further establishing the use of this agent with larger clinical studies, including more diversified patient groups, identifying those patients in everyday clinical practice who could benefit from it is crucial in order to offer enhanced antiplatelet coverage and potentially reduce future thrombotic events. Further trials providing guidance on the identification of such optimal patient characteristics while also exploring novel indications for its use are going to determine the potential benefits of this agent beyond the anti-thrombotic and shape the future suggestions for its clinical use.

## References

[1] Davì G., Patrono C. (2007). Platelet Activation and Atherothrombosis. N. Engl. J. Med..

[2] Angiolillo D.A., Galli M., Collet J.-P., Kastrati A., O’Donoghue M.O. (2022). Antiplatelet Therapy after Percutaneous Coronary Intervention. EuroIntervention.

[3] Gragnano F., Mehran R., Branca M., Franzone A., Baber U., Jang Y., Kimura T., Hahn J.-Y., Zhao Q., Windecker S. (2023). P2Y12 Inhibitor Monotherapy or Dual Antiplatelet Therapy After Complex Percutaneous Coronary Interventions. J. Am. Coll. Cardiol..

[4] Nicolas J., Dangas G., Chiarito M., Pivato C.A., Spirito A., Cao D., Giustino G., Beerkens F., Camaj A., Vogel B. (2023). Efficacy and Safety of P2Y12 Inhibitor Monotherapy after Complex PCI: A Collaborative Systematic Review and Meta-Analysis. Eur. Heart J. Cardiovasc. Pharmacother..

[5] Byrne R.A., Rossello X., Coughlan J.J., Barbato E., Berry C., Chieffo A., Claeys M.J., Dan G.-A., Dweck M.R., Galbraith M. (2023). 2023 ESC Guidelines for the Management of Acute Coronary Syndromes. Eur. Heart J..

[6] De Luca L., Steg P.G., Bhatt D.L., Capodanno D., Angiolillo D.J. (2021). Cangrelor: Clinical Data, Contemporary Use, and Future Perspectives. J. Am. Heart Assoc..

[7] Lawton J.S., Tamis-Holland J.E., Bangalore S., Bates E.R., Beckie T.M., Bischoff J.M., Bittl J.A., Cohen M.G., DiMaio J.M., Don C.W. (2022). 2021 ACC/AHA/SCAI Guideline for Coronary Artery Revascularization. J. Am. Coll. Cardiol..

[8] Ferri N., Corsini A., Bellosta S. (2013). Pharmacology of the New P2Y12 Receptor Inhibitors: Insights on Pharmacokinetic and Pharmacodynamic Properties. Drugs.

[9] Secco G.G., Parisi R., Mirabella F., Fattori R., Genoni G., Agostoni P., De Luca G., Marino P.N., Lupi A., Rognoni A. (2013). P2Y12 Inhibitors: Pharmacologic Mechanism and Clinical Relevance. Cardiovasc. Hematol. Agents Med. Chem..

[10] Remijn J.A., Wu Y.-P., Jeninga E.H., IJsseldijk M.J.W., van Willigen G., de Groot P.G., Sixma J.J., Nurden A.T., Nurden P. (2002). Role of ADP Receptor P2Y _12_ in Platelet Adhesion and Thrombus Formation in Flowing Blood. Arterioscler. Thromb. Vasc. Biol..

[11] Sharis P.J., Cannon C.P., Loscalzo J. (1998). The Antiplatelet Effects of Ticlopidine and Clopidogrel. Ann. Intern. Med..

[12] Damman P., Woudstra P., Kuijt W.J., de Winter R.J., James S.K. (2012). P2Y12 Platelet Inhibition in Clinical Practice. J. Thromb. Thrombolysis.

[13] Akers W.S., Oh J.J., Oestreich J.H., Ferraris S., Wethington M., Steinhubl S.R. (2010). Pharmacokinetics and Pharmacodynamics of a Bolus and Infusion of Cangrelor: A Direct, Parenteral P2Y12 Receptor Antagonist. J. Clin. Pharmacol..

[14] Franchi F., Rollini F., Muñiz-Lozano A., Rae Cho J., Angiolillo D.J. (2013). Cangrelor: A Review on Pharmacology and Clinical Trial Development. Expert. Rev. Cardiovasc. Ther..

[15] Srinivasan S., Mir F., Huang J.-S., Khasawneh F.T., Lam S.C.-T., Le Breton G.C. (2009). The P2Y12 Antagonists, 2-Methylthioadenosine 5’-Monophosphate Triethylammonium Salt and Cangrelor (ARC69931MX), Can Inhibit Human Platelet Aggregation through a Gi-Independent Increase in CAMP Levels. J. Biol. Chem..

[16] Xiang B., Zhang G., Ren H., Sunkara M., Morris A.J., Gartner T.K., Smyth S.S., Li Z. (2012). The P2Y12 Antagonists, 2MeSAMP and Cangrelor, Inhibit Platelet Activation through P2Y12/Gi-Dependent Mechanism. PLoS ONE.

[17] Meadows T.A., Bhatt D.L. (2007). Clinical Aspects of Platelet Inhibitors and Thrombus Formation. Circ. Res..

[18] Lau E.S., Braunwald E., Murphy S.A., Wiviott S.D., Bonaca M.P., Husted S., James S.K., Wallentin L., Clemmensen P., Roe M.T. (2017). Potent P2Y 12 Inhibitors in Men Versus Women. J. Am. Coll. Cardiol..

[19] Storey R.F., Oldroyd K.G., Wilcox R.G. (2001). Open Multicentre Study of the P2T Receptor Antagonist AR-C69931MX Assessing Safety, Tolerability and Activity in Patients with Acute Coronary Syndromes. Thromb. Haemost..

[20] Greenbaum A.B., Grines C.L., Bittl J.A., Becker R.C., Kereiakes D.J., Gilchrist I.C., Clegg J., Stankowski J.E., Grogan D.R., Harrington R.A. (2006). Initial Experience with an Intravenous P2Y12 Platelet Receptor Antagonist in Patients Undergoing Percutaneous Coronary Intervention: Results from a 2-Part, Phase II, Multicenter, Randomized, Placebo- and Active-Controlled Trial. Am. Heart J..

[21] Bhatt D.L., Lincoff A.M., Gibson C.M., Stone G.W., McNulty S., Montalescot G., Kleiman N.S., Goodman S.G., White H.D., Mahaffey K.W. (2009). Intravenous Platelet Blockade with Cangrelor during PCI. N. Engl. J. Med..

[22] Leonardi S., Truffa A.A.M., Neely M.L., Tricoci P., White H.D., Gibson C.M., Wilson M., Stone G.W., Harrington R.A., Bhatt D.L. (2013). A Novel Approach to Systematically Implement the Universal Definition of Myocardial Infarction: Insights from the CHAMPION PLATFORM Trial. Heart.

[23] Harrington R.A., Stone G.W., McNulty S., White H.D., Lincoff A.M., Gibson C.M., Pollack C.V., Montalescot G., Mahaffey K.W., Kleiman N.S. (2009). Platelet Inhibition with Cangrelor in Patients Undergoing PCI. N. Engl. J. Med..

[24] White H.D., Chew D.P., Dauerman H.L., Mahaffey K.W., Gibson C.M., Stone G.W., Gruberg L., Harrington R.A., Bhatt D.L. (2012). Reduced Immediate Ischemic Events with Cangrelor in PCI: A Pooled Analysis of the CHAMPION Trials Using the Universal Definition of Myocardial Infarction. Am. Heart J..

[25] Bhatt D.L., Stone G.W., Mahaffey K.W., Gibson C.M., Steg P.G., Hamm C.W., Price M.J., Leonardi S., Gallup D., Bramucci E. (2013). Effect of Platelet Inhibition with Cangrelor during PCI on Ischemic Events. N. Engl. J. Med..

[26] Abtan J., Steg P.G., Stone G.W., Mahaffey K.W., Gibson C.M., Hamm C.W., Price M.J., Abnousi F., Prats J., Deliargyris E.N. (2016). Efficacy and Safety of Cangrelor in Preventing Periprocedural Complications in Patients With Stable Angina and Acute Coronary Syndromes Undergoing Percutaneous Coronary Intervention. JACC Cardiovasc. Interv..

[27] O’Donoghue M.L., Bhatt D.L., Stone G.W., Steg P.G., Gibson C.M., Hamm C.W., Price M.J., Prats J., Liu T., Deliargyris E.N. (2016). Efficacy and Safety of Cangrelor in Women Versus Men During Percutaneous Coronary Intervention. Circulation.

[28] White H.D., Bhatt D.L., Gibson C.M., Hamm C.W., Mahaffey K.W., Price M.J., Steg P.G., Stone G.W., Cortese B., Wilensky M. (2015). Outcomes With Cangrelor Versus Clopidogrel on a Background of Bivalirudin. JACC Cardiovasc. Interv..

[29] Généreux P., Stone G.W., Harrington R.A., Gibson C.M., Steg P.G., Brener S.J., Angiolillo D.J., Price M.J., Prats J., LaSalle L. (2014). Impact of Intraprocedural Stent Thrombosis During Percutaneous Coronary Intervention. J. Am. Coll. Cardiol..

[30] Steg P.G., Bhatt D.L., Hamm C.W., Stone G.W., Gibson C.M., Mahaffey K.W., Leonardi S., Liu T., Skerjanec S., Day J.R. (2013). Effect of Cangrelor on Periprocedural Outcomes in Percutaneous Coronary Interventions: A Pooled Analysis of Patient-Level Data. Lancet.

[31] Grimfjärd P., Lagerqvist B., Erlinge D., Varenhorst C., James S. (2019). Clinical Use of Cangrelor: Nationwide Experience from the Swedish Coronary Angiography and Angioplasty Registry (SCAAR). Eur. Heart J. Cardiovasc. Pharmacother..

[32] Thim T., Jakobsen L., Jensen R.V., Støttrup N., Eftekhari A., Grove E.L., Larsen S.B., Sørensen J.T., Carstensen S., Amiri S. (2023). Real-World Experience with Cangrelor as Adjuvant to Percutaneous Coronary Intervention: A Single-Centre Observational Study. Cardiol. Res. Pract..

[33] Silverio A., Bellino M., Scudiero F., Attisano T., Baldi C., Catalano A., Centore M., Cesaro A., Di Maio M., Esposito L. (2024). Intravenous Antiplatelet Therapy in Patients with ST-Segment Elevation Myocardial Infarction Undergoing Primary Percutaneous Coronary Intervention. J. Thromb. Thrombolysis.

[34] Steinhubl S.R., Oh J.J., Oestreich J.H., Ferraris S., Charnigo R., Akers W.S. (2008). Transitioning Patients from Cangrelor to Clopidogrel: Pharmacodynamic Evidence of a Competitive Effect. Thromb. Res..

[35] DOVLATOVA N.L., JAKUBOWSKI J.A., SUGIDACHI A., HEPTINSTALL S. (2008). The Reversible P2Y12 Antagonist Cangrelor Influences the Ability of the Active Metabolites of Clopidogrel and Prasugrel to Produce Irreversible Inhibition of Platelet Function. J. Thromb. Haemost..

[36] Dobesh P.P., Oestreich J.H. (2014). Ticagrelor: Pharmacokinetics, Pharmacodynamics, Clinical Efficacy, and Safety. Pharmacother. J. Human. Pharmacol. Drug Ther..

[37] Franchi F., Rollini F., Rivas A., Wali M., Briceno M., Agarwal M., Shaikh Z., Nawaz A., Silva G., Been L. (2019). Platelet Inhibition With Cangrelor and Crushed Ticagrelor in Patients With ST-Segment–Elevation Myocardial Infarction Undergoing Primary Percutaneous Coronary Intervention. Circulation.

[38] Franchi F., Ortega-Paz L., Rollini F., Galli M., Been L., Ghanem G., Shalhoub A., Ossi T., Rivas A., Zhou X. (2023). Cangrelor in Patients With Coronary Artery Disease Pretreated With Ticagrelor. JACC Cardiovasc. Interv..

[39] Rymer J., Alhanti B., Kemp S., Bhatt D.L., Kochar A., Angiolillo D.J., Diaz M., Garratt K.N., Wimmer N.J., Waksman R. (2024). Risk of Bleeding Among Cangrelor-Treated Patients Administered Upstream P2Y12 Inhibitor Therapy: The CAMEO Registry. J. Soc. Cardiovasc. Angiogr. Interv..

[40] De Luca L., Calabrò P., Capranzano P., Di Mario C., Chirillo F., Rolfo C., Menozzi A., Menichelli M., Bolognese L., Musumeci G. (2023). Safety of Cangrelor and Transition to Oral P2Y12 Inhibitors in Patients Undergoing Percutaneous Coronary Intervention: The ARCANGELO Study. Eur. Heart J. Open.

[41] Franchi F., Rollini F., Ortega-Paz L., Been L., Giordano S., Galli M., Ghanem G., Garabedian H., Al Saleh T., Uzunoglu E. (2023). Switching From Cangrelor to Prasugrel in Patients Undergoing Percutaneous Coronary Intervention. JACC Cardiovasc. Interv..

[42] Dimitriadis K., Pyrpyris N., Sakalidis A., Dri E., Iliakis P., Tsioufis P., Tatakis F., Beneki E., Fragkoulis C., Aznaouridis K. (2024). ANOCA Updated: From Pathophysiology to Modern Clinical Practice. Cardiovasc. Revascularization Med..

[43] Pyrpyris N., Dimitriadis K., Iliakis P., Theofilis P., Beneki E., Terentes-Printzios D., Sakalidis A., Antonopoulos A., Aznaouridis K., Tsioufis K. (2024). Hypothermia for Cardioprotection in Acute Coronary Syndrome Patients: From Bench to Bedside. J. Clin. Med..

[44] Moris D., Spartalis M., Spartalis E., Karachaliou G.-S., Karaolanis G.I., Tsourouflis G., Tsilimigras D.I., Tzatzaki E., Theocharis S. (2017). The Role of Reactive Oxygen Species in the Pathophysiology of Cardiovascular Diseases and the Clinical Significance of Myocardial Redox. Ann. Transl. Med..

[45] Luo X., Cai H., Ni J., Bhindi R., Lowe H.C., Chesterman C.N., Khachigian L.M. (2009). C-Jun DNAzymes Inhibit Myocardial Inflammation, ROS Generation, Infarct Size, and Improve Cardiac Function After Ischemia-Reperfusion Injury. Arterioscler. Thromb. Vasc. Biol..

[46] Zhao W., Zhao D., Yan R., Sun Y. (2009). Cardiac Oxidative Stress and Remodeling Following Infarction: Role of NADPH Oxidase. Cardiovasc. Pathol..

[47] Bulluck H., Foin N., Tan J.W., Low A.F., Sezer M., Hausenloy D.J. (2017). Invasive Assessment of the Coronary Microcirculation in Reperfused ST-Segment–Elevation Myocardial Infarction Patients. Circ. Cardiovasc. Interv..

[48] Kelshiker M.A., Seligman H., Howard J.P., Rahman H., Foley M., Nowbar A.N., Rajkumar C.A., Shun-Shin M.J., Ahmad Y., Sen S. (2022). Coronary Flow Reserve and Cardiovascular Outcomes: A Systematic Review and Meta-Analysis. Eur. Heart J..

[49] Aldujeli A., Tsai T., Haq A., Tatarunas V., Knokneris A., Briedis K., Unikas R., Onuma Y., Brilakis E.S., Serruys P.W. (2024). Impact of Coronary Microvascular Dysfunction on Functional Left Ventricular Remodeling and Diastolic Dysfunction. J. Am. Heart Assoc..

[50] Carrick D., Haig C., Ahmed N., Carberry J., Yue May V.T., McEntegart M., Petrie M.C., Eteiba H., Lindsay M., Hood S. (2016). Comparative Prognostic Utility of Indexes of Microvascular Function Alone or in Combination in Patients With an Acute ST-Segment–Elevation Myocardial Infarction. Circulation.

[51] Fearon W.F., Shah M., Ng M., Brinton T., Wilson A., Tremmel J.A., Schnittger I., Lee D.P., Vagelos R.H., Fitzgerald P.J. (2008). Predictive Value of the Index of Microcirculatory Resistance in Patients With ST-Segment Elevation Myocardial Infarction. J. Am. Coll. Cardiol..

[52] Murai T., Yonetsu T., Kanaji Y., Usui E., Hoshino M., Hada M., Hamaya R., Kanno Y., Lee T., Kakuta T. (2018). Prognostic Value of the Index of Microcirculatory Resistance after Percutaneous Coronary Intervention in Patients with Non-ST-segment Elevation Acute Coronary Syndrome. Catheter. Cardiovasc. Interv..

[53] Canu M., Khouri C., Marliere S., Vautrin E., Piliero N., Ormezzano O., Bertrand B., Bouvaist H., Riou L., Djaileb L. (2022). Prognostic Significance of Severe Coronary Microvascular Dysfunction Post-PCI in Patients with STEMI: A Systematic Review and Meta-Analysis. PLoS ONE.

[54] De Maria G.L., Alkhalil M., Wolfrum M., Fahrni G., Borlotti A., Gaughran L., Dawkins S., Langrish J.P., Lucking A.J., Choudhury R.P. (2019). Index of Microcirculatory Resistance as a Tool to Characterize Microvascular Obstruction and to Predict Infarct Size Regression in Patients With STEMI Undergoing Primary PCI. JACC Cardiovasc. Imaging.

[55] El Farissi M., Zimmermann F.M., De Maria G.L., van Royen N., van Leeuwen M.A.H., Carrick D., Carberry J., Wijnbergen I.F., Konijnenberg L.S.F., Hoole S.P. (2023). The Index of Microcirculatory Resistance After Primary PCI. JACC Cardiovasc. Interv..

[56] De Bruyne B., Pijls N.H.J., Gallinoro E., Candreva A., Fournier S., Keulards D.C.J., Sonck J., van’t Veer M., Barbato E., Bartunek J. (2021). Microvascular Resistance Reserve for Assessment of Coronary Microvascular Function. J. Am. Coll. Cardiol..

[57] Tsai T.-Y., Aldujeli A., Haq A., Knokneris A., Briedis K., Hughes D., Unikas R., Renkens M., Revaiah P.C., Tobe A. (2024). The Impact of Microvascular Resistance Reserve on the Outcome of Patients With STEMI. JACC Cardiovasc. Interv..

[58] Liu D.-L., Bao W.-W., Zeng X.-M., Liu X.-T., Zhang Z. (2023). The Effect of Ticagrelor on Myocardial Microcirculation, Cardiac Function, and Adverse Cardiovascular Events in STEMI Patients after PCI. Eur. Rev. Med. Pharmacol. Sci..

[59] Mangiacapra F., Colaiori I., Di Gioia G., Pellicano M., Heyse A., Paolucci L., Peace A., Bartunek J., de Bruyne B., Barbato E. (2024). Effects of Ticagrelor and Prasugrel on Coronary Microcirculation in Elective Percutaneous Coronary Intervention. Heart.

[60] Park S.-D., Lee M.-J., Baek Y.-S., Kwon S.-W., Shin S.-H., Woo S.-I., Kim D.-H., Kwan J., Park K.-S. (2016). Randomised Trial to Compare a Protective Effect of Clopidogrel Versus TIcagrelor on Coronary Microvascular Injury in ST-Segment Elevation Myocardial Infarction (CV-TIME Trial). EuroIntervention.

[61] Park K., Cho Y.-R., Park J.-S., Park T.-H., Kim M.-H., Kim Y.-D. (2019). Comparison of the Effects of Ticagrelor and Clopidogrel on Microvascular Dysfunction in Patients With Acute Coronary Syndrome Using Invasive Physiologic Indices. Circ. Cardiovasc. Interv..

[62] Qiu X., Li X., Fu K., Chen W., Chen W. (2023). The Effect of Ticagrelor on Coronary Microvascular Function after PCI in Patients with ACS Compared to Clopidogrel: A Systematic Review and Meta-Analysis. PLoS ONE.

[63] Li Y., Ye Z., Guo Z., Xie E., Wang M., Zhao X., Liu M., Li P., Yu C., Gao Y. (2023). Ticagrelor vs. Clopidogrel for Coronary Microvascular Dysfunction in Patients with STEMI: A Meta-Analysis of Randomized Controlled Trials. Front. Cardiovasc. Med..

[64] van Leeuwen M.A.H., van der Hoeven N.W., Janssens G.N., Everaars H., Nap A., Lemkes J.S., de Waard G.A., van de Ven P.M., van Rossum A.C., ten Cate T.J.F. (2019). Evaluation of Microvascular Injury in Revascularized Patients With ST-Segment–Elevation Myocardial Infarction Treated With Ticagrelor Versus Prasugrel. Circulation.

[65] Bonello L., Laine M., Kipson N., Mancini J., Helal O., Fromonot J., Gariboldi V., Condo J., Thuny F., Frere C. (2014). Ticagrelor Increases Adenosine Plasma Concentration in Patients With an Acute Coronary Syndrome. J. Am. Coll. Cardiol..

[66] Dimitriadis K., Theofilis P., Koutsopoulos G., Pyrpyris N., Beneki E., Tatakis F., Tsioufis P., Chrysohoou C., Fragkoulis C., Tsioufis K. (2024). The Role of Coronary Microcirculation in Heart Failure with Preserved Ejection Fraction: An Unceasing Odyssey. Heart Fail. Rev..

[67] Ganbaatar B., Fukuda D., Salim H.M., Nishimoto S., Tanaka K., Higashikuni Y., Hirata Y., Yagi S., Soeki T., Sata M. (2018). Ticagrelor, a P2Y12 Antagonist, Attenuates Vascular Dysfunction and Inhibits Atherogenesis in Apolipoprotein-E-Deficient Mice. Atherosclerosis.

[68] Sexton T.R., Zhang G., Macaulay T.E., Callahan L.A., Charnigo R., Vsevolozhskaya O.A., Li Z., Smyth S. (2018). Ticagrelor Reduces Thromboinflammatory Markers in Patients With Pneumonia. JACC Basic. Transl. Sci..

[69] Hagiwara S., Iwasaka H., Hasegawa A., Oyama M., Imatomi R., Uchida T., Noguchi T. (2011). Adenosine Diphosphate Receptor Antagonist Clopidogrel Sulfate Attenuates LPS-Induced Systemic Inflammation in a Rat Model. Shock.

[70] Mansour A., Bachelot-Loza C., Nesseler N., Gaussem P., Gouin-Thibault I. (2020). P2Y12 Inhibition beyond Thrombosis: Effects on Inflammation. Int. J. Mol. Sci..

[71] Wang X., Han X., Li M., Han Y., Zhang Y., Zhao S., Li Y. (2018). Ticagrelor Protects against AngII-Induced Endothelial Dysfunction by Alleviating Endoplasmic Reticulum Stress. Microvasc. Res..

[72] Olgar Y., Tuncay E., Billur D., Durak A., Ozdemir S., Turan B. (2020). Ticagrelor Reverses the Mitochondrial Dysfunction through Preventing Accumulated Autophagosomes-Dependent Apoptosis and ER Stress in Insulin-Resistant H9c2 Myocytes. Mol. Cell Biochem..

[73] Jeong H.S., Hong S.J., Cho S.-A., Kim J.-H., Cho J.Y., Lee S.H., Joo H.J., Park J.H., Yu C.W., Lim D.-S. (2017). Comparison of Ticagrelor Versus Prasugrel for Inflammation, Vascular Function, and Circulating Endothelial Progenitor Cells in Diabetic Patients With Non–ST-Segment Elevation Acute Coronary Syndrome Requiring Coronary Stenting. JACC Cardiovasc. Interv..

[74] Schnorbus B., Daiber A., Jurk K., Warnke S., Koenig J., Lackner K.J., Münzel T., Gori T. (2020). Effects of Clopidogrel vs. Prasugrel vs. Ticagrelor on Endothelial Function, Inflammatory Parameters, and Platelet Function in Patients with Acute Coronary Syndrome Undergoing Coronary Artery Stenting: A Randomized, Blinded, Parallel Study. Eur. Heart J..

[75] Ariotti S., Ortega-Paz L., van Leeuwen M., Brugaletta S., Leonardi S., Akkerhuis K.M., Rimoldi S.F., Janssens G., Gianni U., van den Berge J.C. (2018). Effects of Ticagrelor, Prasugrel, or Clopidogrel on Endothelial Function and Other Vascular Biomarkers. JACC Cardiovasc. Interv..

[76] Lim S., Choo E.H., Kim C.J., Choi I.J., Lee K.Y., Hwang B.-H., Lee J.-M., Chung W.S., Chang K. (2019). Ticagrelor Does Not Improve Endothelial Dysfunction in Stable Survivors of Acute Coronary Syndrome. J. Cardiovasc. Pharmacol. Ther..

[77] Guan B., Zhao L., Ma D., Fan Y., Zhang H., Wang A., Xu H. (2022). The Effect of Ticagrelor on Endothelial Function Compared to Prasugrel, Clopidogrel, and Placebo: A Systematic Review and Meta-Analysis. Front. Cardiovasc. Med..

[78] Yang X.-M., Liu Y., Cui L., Yang X., Liu Y., Tandon N., Kambayashi J., Downey J.M., Cohen M.V. (2013). Platelet P2Y _12_ Blockers Confer Direct Postconditioning-Like Protection in Reperfused Rabbit Hearts. J. Cardiovasc. Pharmacol. Ther..

[79] Yang X.-M., Liu Y., Cui L., Yang X., Liu Y., Tandon N., Kambayashi J., Downey J.M., Cohen M.V. (2013). Two Classes of Anti-Platelet Drugs Reduce Anatomical Infarct Size in Monkey Hearts. Cardiovasc. Drugs Ther..

[80] Cohen M.V., Yang X.-M., White J., Yellon D.M., Bell R.M., Downey J.M. (2016). Cangrelor-Mediated Cardioprotection Requires Platelets and Sphingosine Phosphorylation. Cardiovasc. Drugs Ther..

[81] Ubaid S., Ford T.J., Berry C., Murray H.M., Wrigley B., Khan N., Thomas M.R., Armesilla A.L., Townend J.N., Khogali S.S. (2019). Cangrelor versus Ticagrelor in Patients Treated with Primary Percutaneous Coronary Intervention: Impact on Platelet Activity, Myocardial Microvascular Function and Infarct Size: A Randomized Controlled Trial. Thromb. Haemost..

[82] Bulluck H., Chong J.H., Bryant J., Annathurai A., Chai P., Chan M., Chawla A., Chin C.Y., Chung Y.-C., Gao F. (2024). Effect of Cangrelor on Infarct Size in ST-Segment–Elevation Myocardial Infarction Treated by Primary Percutaneous Coronary Intervention: A Randomized Controlled Trial (The PITRI Trial). Circulation.

[83] Droppa M., Vaduganathan M., Venkateswaran R.V., Singh A., Szumita P.M., Roberts R.J., Qamar A., Hack L., Rath D., Gawaz M. (2019). Cangrelor in Cardiogenic Shock and after Cardiopulmonary Resuscitation: A Global, Multicenter, Matched Pair Analysis with Oral P2Y12 Inhibition from the IABP-SHOCK II Trial. Resuscitation.

[84] RYMER J., PICHAN C., PAGE C., ALHANTI B., BHATT D.L., KOCHAR A., ANGIOLILLO D.J., DIAZ M., WIMMER N.J., WAKSMAN R. (2024). The Use of Cangrelor in Cardiogenic Shock: Insights from the CAMEO Registry. J. Card. Fail..

[85] Katz A., Lewis T.C., Arnouk S., Altshuler D., Papadopoulos J., Toy B., Smith D.E., Merchan C. (2021). Clinical Use of Cangrelor After Percutaneous Coronary Intervention in Patients Requiring Mechanical Circulatory Support. Ann. Pharmacother..

[86] Kordis P., Bozic Mijovski M., Berden J., Steblovnik K., Blinc A., Noc M. (2023). Cangrelor for Comatose Survivors of Out-of-Hospital Cardiac Arrest Undergoing Percutaneous Coronary Intervention: The CANGRELOR-OHCA Study. EuroIntervention.

[87] Zeymer U., Lober C., Richter S., Olivier C.B., Huber K., Haring B., Schwimmbeck P., Andrassy M., Akin I., Cuneo A. (2023). Cangrelor in Patients with Percutaneous Coronary Intervention for Acute Myocardial Infarction after Cardiac Arrest and/or with Cardiogenic Shock. Eur. Heart J. Acute Cardiovasc. Care.

[88] Thiele H., Akin I., Sandri M., Fuernau G., de Waha S., Meyer-Saraei R., Nordbeck P., Geisler T., Landmesser U., Skurk C. (2017). PCI Strategies in Patients with Acute Myocardial Infarction and Cardiogenic Shock. N. Engl. J. Med..

[89] D’Andria Ursoleo J., Baldetti L., Pieri M., Nardelli P., Altizio S., Ajello S., Scandroglio A.M. (2024). Anti-Platelet Therapy with Cangrelor in Cardiogenic Shock Patients: A Systematic Review and Single-Arm Meta-Analysis. Medicina.

[90] Motovska Z., Hlinomaz O., Mrozek J., Kala P., Geisler T., Hromadka M., Akin I., Precek J., Kettner J., Cervinka P. (2024). Cangrelor versus Crushed Ticagrelor in Patients with Acute Myocardial Infarction and Cardiogenic Shock: Rationale and Design of the Randomised, Double-Blind DAPT-SHOCK-AMI Trial. EuroIntervention.

[91] Angiolillo D.J., Firstenberg M.S., Price M.J., Tummala P.E., Hutyra M., Welsby I.J., Voeltz M.D., Chandna H., Ramaiah C., Brtko M. (2012). Bridging Antiplatelet Therapy With Cangrelor in Patients Undergoing Cardiac Surgery. JAMA.

[92] Rossini R., Masiero G., Fruttero C., Passamonti E., Calvaruso E., Cecconi M., Carlucci C., Mojoli M., Guido P., Talanas G. (2020). Antiplatelet Therapy with Cangrelor in Patients Undergoing Surgery after Coronary Stent Implantation: A Real-World Bridging Protocol Experience. TH Open.

[93] Shrestha B., Katz D., Kelley J., Menzies D., Hong M.K. (2022). Cangrelor in STEMI as a Bridge to CABG- a Mini-Case Series. Am. Heart J. Plus Cardiol. Res. Pract..

[94] Bruck C., Jafar O., Prats J., Bhatt D., Jafar Z. (2018). A Novel Bridging Strategy for Patients Undergoing Emergent Non-Cardiac Surgery with a Recent Coronary Stent. Cardiol. Ther..

[95] Fialho I., Augusto J.B., Fevereiro S., Santos M.B., Baptista S.B., Roque D. (2022). Cangrelor as Antiplatelet Bridging Therapy in Non-Cardiac Surgery after Percutaneous Coronary Intervention–First-Time Use in Portugal. Rev. Port. Cardiol..

[96] Benenati S., Gragnano F., Scalamera R., Bertero E., Capolongo A., De Sio V., Musumeci G., Annibali G., Campagnuolo S., Galasso G. (2022). 330 Intravenous cangrelor infusion in high bleeding risk patients undergoing percutaneous coronary intervention: Preliminary results of the icarus registry. Eur. Heart J. Suppl..

[97] Chaturvedi A., Creechan P., Hill A., Rappaport H., Cellamare M., Sawant V., Zhang C., Abusnina W., Haberman D., Chitturi K. (2024). TCT-174 High Bleeding Risk and Outcomes in Acute Coronary Syndrome After Percutaneous Coronary Intervention With Versus Without Cangrelor. J. Am. Coll. Cardiol..

